# Exploring a Chemotactic Role for EVs from Progenitor Cell Populations of Human Exfoliated Deciduous Teeth for Promoting Migration of Naïve BMSCs in Bone Repair Process

**DOI:** 10.1155/2021/6681771

**Published:** 2021-03-17

**Authors:** Lin Luo, Steven J. Avery, Rachel J. Waddington

**Affiliations:** ^1^Department of Oral and Biomedical Sciences, School of Dentistry, College of Biomedical and Life Sciences, Cardiff University, Cardiff, UK; ^2^Department of Prosthodontics, School of Stomatology, China Medical University, Shenyang, China

## Abstract

Mobilization of naïve bone marrow mesenchymal stromal cells (BMSCs) is crucial to desired bone regeneration in both orthopedic and dental contexts. In such conditions, mesenchymal progenitor cell populations from human exfoliated deciduous teeth (SHEDs) present advantageous multipotent properties with easy accessibility which makes them a good candidate in both bone and periodontal tissue regeneration. Extracellular vesicles (EVs) are a functional membranous structure which could participate in multiple cell interactions and imitate the biological functions of their parenting cells largely. To assess their ability to mobilize naïve BMSCs in the bone repair process, Nanosight Tracking Analysis (NTA) and Enzyme-Linked Immunosorbent Assays (ELISA) were performed to illustrate the composition and functional contents of EV samples derived from SHEDs with different culturing time (24 h, 48 h, and 72 h). Afterwards, the Boyden chamber assay was performed to compare their capacity for mobilizing naïve BMSCs. One-way analysis of variance (ANOVA) with a post hoc Turkey test was performed for statistical analysis. SHEDs-derived EVs collected from 24 h, 48 h, and 72 h time points, namely, EV24, EV48, and EV72, were mainly secreted as exosomes and tended to reform into smaller size as a result of sonication indicated by NTA results. Moreover, different EV groups were found to be abundant with multiple growth factors including transforming growth factor-*β*1 (TGF-*β*1), platelet-derived growth factor (PDGF), insulin-like growth factor-1 (IGF-1), and fibroblast growth factor-2 (FGF-2) given the detections through ELISA. Boyden chamber assays implied the migratory efficiency of BMSCs driven by EVs at varying concentrations. However, the results showed that migration of BMSCs driven by different EV groups was not statistically significant even with chemotactic factors contained (*P* > 0.05). Taken together, these data suggest that EVs derived from SHEDs are secreted in functional forms and present a potential of mobilizing naïve BMSCs, which may propose their relevance in assisting bone regeneration.

## 1. Introduction

Bone loss faces numerous challenges under diseased situations in both orthopedic and dental contexts. Autografts have been recognized as the “gold standard” treatment for bone defects; however, bone harvest volume is largely limited and donor site morbidity is also reported [1, 2]. In oral tissue loss scenarios, dental prostheses and implants can only relieve the clinical symptoms but not impact the loss of bone and the surrounding tissue. The process of natural bone repair is well organized and precisely controlled by numerous molecular signals. When a bone fracture occurs, a series of functional factors including platelet-derived growth factor (PDGF), transforming growth factor-*β*1 (TGF-*β*1), insulin-like growth factors (IGFs), and fibroblast growth factor-2 (FGF-2) is released into the extracellular space at the fracture site [3–5]. These factors collectively mediate the mobilization of bone marrow stromal cells (BMSCs), which proliferate and subsequently differentiate into osteoblasts to contribute to new bone formation. Stem cell transplantation has been shown to be effective for promoting the regenerative potential of bone in cases of nonunion fractures [6]. Nonetheless, stem cell transplantation presents with caveats such as immunosuppression and a depletion of oxygen and nutrients to the microenvironment which makes it harsh for the naïve progenitors to migrate through to the injured area [7]. Therefore, the mobilization of naïve mesenchymal stromal cells (MSCs) to target the injury site would rescue the insufficiency of resident BMSCs and contribute to bone repair [8–10]. BMSCs are recognized multipotent cells, and their reparative performance relies on the mobilization and migration to target sites [11]. Their role in tissue regeneration relies on the paracrine effects and directed differentiation, which is dependent on the targeting migration to the relevant tissue site. It has been noticed that bone marrow-derived multipotent progenitor cells can produce therapeutic effects in a paracrine manner through secretion of factors, rather than engrafting or differentiating [12].

Regarding factors secreted, extracellular vesicles (EVs) are gaining attention presently as they play a role in transporting cargos of proteins and nucleic acids, namely, cell messenger during biological processes. EVs are existing as heterogeneous populations which can be characterized as exosomes (30-100 nm), microvesicles (100-1000 nm), and apoptotic bodies (50 nm-2 *μ*m) depending on the size and function according to definitions by the International Society of the Extracellular Vesicles [13]. Studies have shown that EVs are implicated in both physiological and pathological processes, including stimulating specific and nonspecific immune responses [14], inhibiting tumor growth [15], or drug delivery [16]. Exogenous EVs derived from BMSCs can be internalized by endogenous skeletal cells, thus initiating the differentiation of progenitor bone cells [17]. Together with three-dimensional (3D) scaffolds, EVs have been applied in bone engineering to repair large-scale bone defects. It has been reported that engineered 3D polylactic acid (PLA) scaffolds combined with human gingival stem cells and EVs could produce enhanced bone and extracellular matrix formation [18], which implied that EVs could boost bone repair through internalization within the microenvironment and transporting functional cargos. EVs are also known as functional vesicles with a performance of imitating their parent cells [7]. As oral-derived stem cells showed emerging benefits on stem cell therapy with easy accessibility and multiple differentiation properties, mesenchymal progenitor cell populations from human exfoliated deciduous teeth (SHEDs) have been recognized as multipotent stem cells with osteogenic and odontogenic potential, which could be a significant origin of EVs as SHEDs could be obtained through a technique that is painless and convenient with greater proliferative capacity compared to dental pulp stem cells (DPSCs), which is advantageous to repair bone and periodontal tissues [19].

While these reports indicate the potential for EVs to assist in driving osteogenic processes, knowledge of the mechanisms for their role in bone repair is currently limited. Therefore, we aimed to explore the potential role of EVs derived from SHEDs in mobilizing BMSCs through detecting the chemoattractant factors in EVs and the migration efficiency produced by EVs. Our null hypothesis states that EVs are unable to influence the migration of BMSCs. Confirming a chemoattractant role for these EVs may further demonstrate their role in the bone repair process and potentially be used to alleviate the clinical implications associated with current treatment regimens.

## 2. Materials and Methods

### 2.1. EV-Depleted Medium Generation and SHED Cell Culture

SHEDs were a generous gift from BioEden (BioEden, TX, USA). Cell culture medium was an EV-depleted general medium, composed of Mesenchymal Stem Cell Basal Media (Lonza, UK) supplemented with 15% EV-depleted Fetal Bovine Serum (FBS) (Invitrogen, UK), 1% L-ascorbate 2-phosphate (Sigma-Aldrich, Poole, UK), 1% L-glutamine (Invitrogen, UK), and 1% Antibiotic-Antimycotic (Invitrogen, UK). FBS was inactivated at 59.5°C for 30 min, followed by ultracentrifugation at 100,000 g for 18 h at 4°C (Sorvall Discovery 100SE, UK) and filtration of the resulting supernatant through a 0.2 *μ*m Nalgene™ Rapid-Flow™ filter (Thermo Fisher Scientific, UK) to obtain EV-depleted FBS. Upon thawing, SHEDs were seeded into T175 culture flasks (Sarstedt, Germany) at 10,000 cells/cm^2^ and incubated at 37°C, in 5% CO_2_ and 95% humidity, with medium changes performed every 2-3 days.

### 2.2. EV Conditioned Medium (CM) Collection

When SHEDs reached 80%-90% confluency, culture media were aspirated and replaced with 20 mL fresh culture media. Following this, culture media were collected after 24 h, 48 h, and 72 h, which were regarded as extracellular vesicle culture media (EVCM). Three flasks of EVCM were collected for each group. EVCM underwent a sequential centrifugation process of supernatants to purify EVs (400 g for 5 min, 2,000 g for 15 min, 10,000 g for 30 min, and 100,000 g for 90 min). The resulting EV pellet after the final centrifugation step was resuspended in 5 mL of MEM (containing ribonucleosides and deoxyribonucleosides (Thermo Fisher Scientific, UK)) prior to analyses and utilization in this study, with EVs collected after 24 h, 48 h, and 72 h denoted as EV24, EV48, and EV72, respectively. The supernatant from the final centrifugation step for each EV group was retained for analysis and referred to as top fraction (TF). EVs and TFs were stored at -80°C prior to further analysis.

### 2.3. EV Sonication

1 mL of each EV group was maintained on ice and sonicated (BRANSON Ultrasonics, USA) at maximum intensity for 5 s, three times, with 5 s intervals. EVs with different culture times after sonication were denoted as EV24 SO, EV48 SO, and EV72 SO.

### 2.4. Nanoparticle Tracking Analysis (NTA)

A nanoparticle analysis system (NanoSight LM10 HS microscope, NanoSight Ltd., Amesbury, UK) was used to analyze the abundance and the size of EVs according to previously described protocols [20, 21], corrected with *α*-MEM (Thermo Fisher Scientific, UK) as reference diluent. Samples were thawed and diluted in deionized distilled water at a dilution of 1 : 100 to achieve an approximated concentration between 10^6^ and 109 particles/mL to be analyzed. The particles were detected using a 638 nm laser light, with 6 replicates performed per analysis group. EVs were analyzed using Nanoparticle Tracking Analysis 2.3 analytical software.

### 2.5. Chemotactic Factor Detection

#### 2.5.1. Enzyme-Linked Immunosorbent Assay (ELISA)

Growth factors were detected in EV samples through commercially available kits, following manufacturer protocols: Human TGF-*β*1 Platinum ELISA kit (eBioscience, UK); PDGF-BB and FGF-2 using respective Human Mini ELISA kits (PeproTech, UK); Human IGF-1 Quantikine® ELISA kit (R&D Systems, UK). Assays were performed in duplicate on three separate occasions.

#### 2.5.2. BMSC Culture and Fibronectin Selection

BMSCs were commercially obtained (Lonza, UK), and fibronectin-adherent BMSCs (FNA-BMSCs) were acquired as previously described by Lee et al. [22]. FNA-BMSCs were plated into tissue culture flasks (Sarstedt, Germany) at 5,000 cells/cm^2^ in basal medium *α*-MEM supplemented with 10% FBS, 1% L-ascorbate 2-phosphate (Sigma-Aldrich, Poole, UK), and 1% Antibiotic-Antimycotic (Invitrogen, UK). FNA-BMSCs were cultured at 37°C, 5% CO_2_ with media changed every 2-3 days.

#### 2.5.3. Cell Migration Assay

The potential for SHED-derived EVs to stimulate migration of FNA-BMSCs was performed using a Boyden chamber assay, as previously described [23]. EV concentration matched with the concentration of TGF-*β*1 in 0.1 *μ*g/mL of demineralized dentin matrix (DDM), which has been shown to effectively stimulate FNA-BMSC migration [23], denoted as 1x. A tenfold lower concentration was also used (denoted as 0.1x). Assays were performed in triplicate and on three separate occasions.

### 2.6. Statistical Analysis

For all assays performed above, triplicate samples were analyzed for each sample group (unless otherwise mentioned), and assays were repeated on three separate occasions. Statistical analyses to determine the analysis of variance between sample groups were performed by one-way ANOVA with a post hoc Tukey test using GraphPad Prism 8 software (GraphPad, La Jolla, CA, USA). *P* < 0.05 was regarded as statistically significant.

## 3. Results

### 3.1. EV Analysis Using NTA

**Figure 1:**
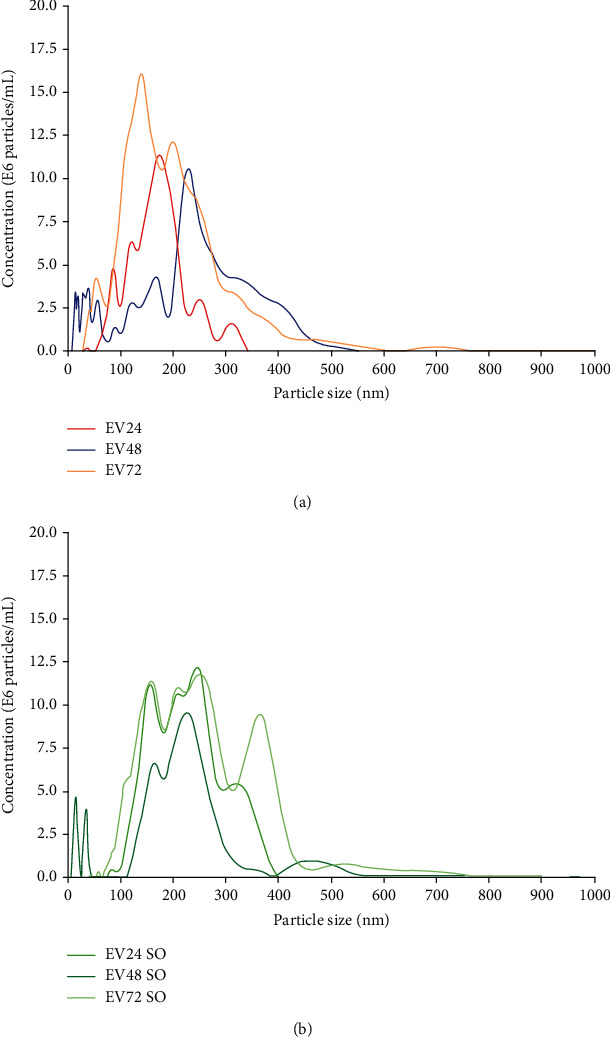
(a) NTA results demonstrating the particle size distribution and concentrations of EVs in each group. Peak concentrations were at particle sizes of approximately 179 nm, 255 nm, and 137 nm for EV24, EV48, and EV72 groups, respectively. (b) NTA results demonstrating the particle size distribution and concentrations of EVs in each group after sonication. Peaks appear to be much less defined compared to respective native EVs.

NTA was performed to analyze the particle size and concentration [20]. Figures 1(a) and 1(b) illustrate the concentrations of particles and the particle size, corrected to EV-depleted media in all groups. All EV groups showed a relative particle concentration. Moreover, the particle size of all of the groups was limited to 1000 nm, which matched the particle size composition of EVs [24]. For both unsonicated (EV) and sonicated (SO) groups, the highest concentrations of particles were for EV72 and EV72 SO groups. The peak concentration of particles in each group varied in particle size. The particle size was predominantly between 50 and 120 nm [25], which refers to an exosome size. The profile of size distribution was different for each time point. For the EV groups, the particle concentration peaked around 150-180 nm for EV24, while EV48 and EV72 peaked at 250-280 nm and 210-230 nm, respectively. For the SO groups, the distribution of particles was changed as a result of sonication in all EV groups, where the number of particles with the size over 300 nm was reduced while that with the size between 100 nm and 300 nm was increased.

### 3.2. Chemotactic Factor Quantification Using ELISA

**Figure 2:**
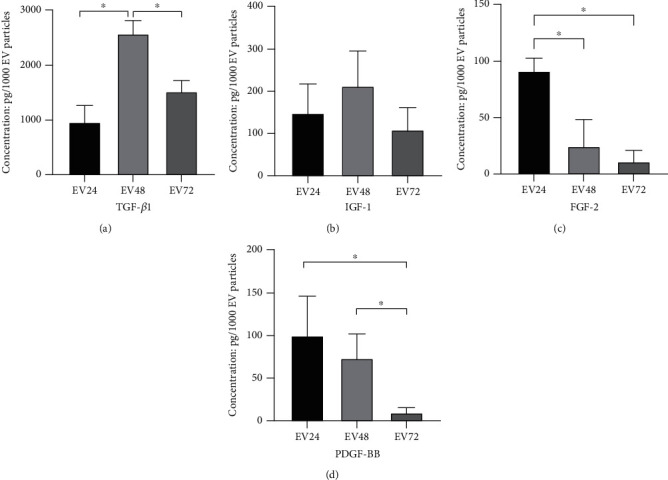
ELISA analyses of growth factors contained in EV groups, standardized per 1,000 particles. TGF-*β*1 concentration was significantly higher for the EV48 group compared to EV24 and EV72 groups (a). IGF-1 concentration was unchanged between EV groups (b). FGF-2 concentration was shown to be higher in EV24 groups compared to EV48 and EV72 groups (c). The concentration of PDGF-BB was significantly lower in the EV72 group compared to EV24 and EV48 groups (d). The data are presented as mean ± SD (*n* = 3). ^∗^*P* < 0.05.

The EV samples were assayed for chemotactic factor concentration by ELISA after sonication, in order to release their cargos for analyses. Figures 2(a)–2(d) show the different concentrations of TGF-*β*1, IGF-1, FGF-2, and PDGF-BB contained in EV24, EV48, and EV72 groups. The concentrations were corrected relative to -MEM. For TGF-*β*1, Figure 2(a) shows that the highest concentration was present in EV48 at 2,556 pg/10^3^ particles, which was significantly higher than both EV24 and EV72 samples (*P* < 0.05). There was no significant difference of TGF-*β*1 concentration between EV24 and EV72 samples (*P* > 0.05). For IGF-1, Figure 2(b) shows that EV24 and EV48 contain slightly higher IGF-1 than EV72 where there were no significant differences in concentration between EV24, EV48, and EV 72 groups (*P* > 0.05). For PDGF-BB, EV24 (79 pg/10^3^ particles) and EV48 (48 pg/10^3^ particles) groups both demonstrated a significantly greater concentration than EV72 (8 pg/10^3^ particles) (*P* < 0.05). However, no difference in concentration was observed between EV24 and EV48 groups (*P* > 0.05) (Figure 2(c)). For FGF-2, the highest concentration (90 pg/10^3^ particles) was present in EV24 samples, which was significantly higher than both EV48 and EV72 samples (*P* < 0.05) (Figure 2(d)). There was no significant difference in FGF-2 concentration between EV48 and EV72 samples (*P* > 0.05).

### 3.3. Cell Migration Analysis

**Figure 3:**
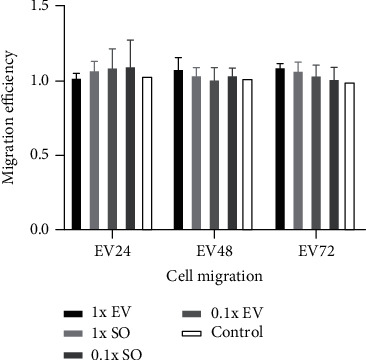
Cell migration efficiency of FNA-BMSCs induced by different EV groups, relative to controls. No significant differences were observed between groups. All of the data are presented as mean ± SD.

Boyden chamber assays were performed to detect the migration potential of FNA-BMSCs driven by unsonicated and sonicated EVs, based on the 1x and 0.1x concentration of TGF-*β*1 required for BMSC migration (15.6 pg/mL and 1.56 pg/mL, respectively), as demonstrated previously [23]. EV groups were diluted into 1x and 0.1x with or without sonication in EV24, EV48, and EV72 groups. Serum-free media were used as a negative control.  Figure 3 shows the different fluorescent signals which resembled the relative number of cells migrated through the collagen-coated membranes. The different migratory activities were detected by comparing the migration relative to the negative control. The migration efficiency among groups was slightly improved driven by the EVs compared to control, but it was not statistically significant (*P* > 0.05). SO groups demonstrated slightly better chemotactic effects than unsonicated groups in EV24 with (0.1x) or without (1x) dilution, while EVs without dilution (1x) showed slightly better chemotactic efficiency in EV48 and EV72 groups towards FNA-BMSCs.

## 4. Discussion

EVs have been described as important mediators during critical functional and biological processes among cells in disease contexts or tissue repair [26, 27]. Given the property of imitating parent cells [28], the origin of EVs should be taken into consideration. In the periodontal repair context, SHEDs were previously explored with both bone and periodontal tissue repair capacity. In an in vitro model, SHEDs present great biological responses including cell viability and morphology on silk fibroin scaffolds [29], which prepared SHEDs a promising candidate for tissue engineering. In a bone defect model, SHED-derived conditioned media (SHED-CM) and SHEDs were transplanted into the defect area and the osteogenic outcomes were compared. SHED-CM showed better bone regeneration results with improved bone mineralization and angiogenic potential [30]. Multiple cytokines were also detected, aiming to elucidate the functional factors contained in SHED-CM. It was reported that SHED-CM was abundant in bone morphogenetic protein-2 (BMP-2), BMP-4, and osteoprotegerin (OPG) and presented an anti-inflammation performance [30, 31], suggesting that SHEDs serve as a promising candidate beneficial to osteogenesis in a paracrine manner.

As mobilizing BMSCs to the target site presents a critical role in bone repair, in the present study, we hoped to illustrate how SHED-derived EVs perform for driving naïve MSC migration where a series of detections was performed. The NTA results provided an overall composition in terms of the number and size of SHED-derived EVs. Most of the EVs were in a diameter of 50-200 nm, suggesting that SHED-derived EVs were mainly secreted as exosomes. The NTA results also provided information regarding the sonication effects on EVs as the EVs are membrane vesicles that need to be disrupted to release contents. With the sonication performed, the mean particle size was reduced and the particles demonstrated a wider size distribution. This method contributing to content release of EVs was also mentioned by Haque et al. [32]. It was suggested that different types and concentrations of the detergents would cause low disruption and reformation of the particles [32]; hence, sonication was applied to disrupt EVs in this study.

To detect the chemotactic ability of SHED-derived EVs, the chemotactic factors were investigated in the EV groups. It is acknowledged that TGF-*β*1 is one of the most important factors to drive MSC migration as well as IGF-1 [4, 33]. Both factors were found in all EV groups in our study. In addition, the concentration of PDGF-BB and FGF-2 was also detected mostly in the EV24 group, compared to the EV48 and EV72 groups. As culturing time increased, EVs underwent subsequent uptake which possibly gave rise to their reduction in the concentration of growth factors in EV48 and EV72 groups [13]. As the potential of EVs to drive MSC migration has been mentioned in both bone repair and wound healing scenarios [34, 35], cell migration assays were performed to determine the chemotactic ability of SHED-derived EVs towards BMSCs. In our research, the EV24 group showed slight but not significantly different chemotactic potential which was consistent with their wider composition of contents, as EV24 are abundant with growth factors with more diversity. However, the recruitment of MSCs was not significantly different among the groups according to the assays. This indicates that the chemotactic factors contained in EVs were still not sufficient to drive migration in our study. According to the previous work by our group, the migration process could be driven by DDM with more bioactive contents, which also confirmed that the migratory process of MSCs requires a cooperating work of multiple factors [23]. In our previous study, DDM in its 0.1 *μ*g/mL concentration induced better cell migration compared to 10x or 100x higher concentrations [23], which gives the idea that EVs require optimization to more potent concentrations, and could be a potential avenue for investigation in future work. Another potential explanation is that the presence of additional matrix components in DDM, which may not be present in EVs, enhanced the migratory potential of chemotactic factors at comparable concentrations [23]. Meanwhile, it was suggested that the MSCs were more likely to migrate when there is a stimulus present other than in a quiescent state [36]. In fact, to mobilize more naïve MSCs to target sites, abundant efforts were made in the past. It was reviewed that multiple chemoattractants participate in the recruitment of MSCs in vitro [37]. On the other hand, it is reported that the gradient sensitivity of the established assay would be too small to drive obvious migration performance [34]. The migration ability of MSCs also varies on properties including senescence of stem cells and condition of the local stem cell niche [38]. In such regard, the established assays for detecting the chemotactic role of EVs derived from SHEDs should be optimized in aspects of concentration, cooperating with other chemoattractants, and passage of BMSCs used, which are also the limitations in this research. Even though the EVs carry functional cargos in our analysis, how they interact with cells in the bone repair microenvironment inside the body still requires further detection.

SHED-derived EVs abundant in exosomes contain various cargos including chemotactic factors, giving rise to the possibility to drive cell migration. This offers a chance for SHED-derived EVs to mobilize more progenitor cells in a bone repair context, and efforts could be made to promote the bone healing process by stimulating the secretion of EV cargos where EVs could function as a vehicle for a therapeutic purpose [39]. Even these factors seem to be insufficient to drive the migration of naïve BMSCs which might be because multiple growth factor populations would work collectively to mediate cell migration. Taken together, our results illustrated the potential of EVs originated from SHEDs to serve as attractants of BMSCs, which was evident by heterogenous growth factors contained whereas the ideal concentration of EVs still required optimization in the future, particularly in relation to other constituents which may or may not be present in EV cargos, that influence the biological effects of chemotactic factors.

Overall, our work contributes to a better understanding of mobilizing BMSCs in a clinical context and the interactions of EVs from SHEDs in the naïve bone repair microenvironment which may offer new treatment options for hindered bone repair.

## Data Availability

The underlying data are available on request.
